# Identification of Molecular Subtypes and a Novel Prognostic Model of Sepsis Based on Ferroptosis-Associated Gene Signature

**DOI:** 10.3390/biom12101479

**Published:** 2022-10-14

**Authors:** Hang Yang, Yanhui Cui, Wenzhong Peng, Fei Zhu, Shiyang Ma, Mingjun Rao, Peipei Zhang, Jie Chen, Pinhua Pan

**Affiliations:** 1 Department of Respiratory Medicine, National Key Clinical Specialty, Branch of National Clinical Research Center for Respiratory Disease, Xiangya Hospital, Central South University, Changsha 410008, China; 2 Center of Respiratory Medicine, Xiangya Hospital, Central South University, Changsha 410008, China; 3 Clinical Research Center for Respiratory Diseases in Hunan Province, Changsha 410008, China; 4 Hunan Engineering Research Center for Intelligent Diagnosis and Treatment of Respiratory Disease, Changsha 410008, China; 5 National Clinical Research Center for Geriatric Disorders, Xiangya Hospital, Changsha 410008, China; 6 Department of Infection Control Center of Xiangya Hospital, Central South University, Changsha 410008, China

**Keywords:** sepsis, ferroptosis, molecular subtype, prognosis

## Abstract

Ferroptosis has recently been associated with immunological changes in sepsis. However, the clinical significance of ferroptosis-associated genes (FAGs) remains unknown. In this paper, a FAG-based prognostic model was constructed for sepsis patients using an integrated machine learning approach. The prognosis model was composed of 14 FAGs that classify the patients as high or low risk. Based on immunological study, it was found that the immune status differed between the high-risk and low-risk clusters. Cox regression analysis revealed that FAGs were independent risk factors for the overall survival of sepsis patients. ROC curves and nomograms revealed that the FAG-based model was robust for prognosis prediction. Lastly, NEDD4L was identified from the 14 FAGs as a potential hub gene for sepsis prediction.

## 1. Introduction

Sepsis, caused by dysregulation of the host response, can result in life-threatening organ dysfunction [1], and has become the leading cause of mortality for patients in the intensive care unit [2]. Approximately 50 million cases of sepsis were reported globally in 2017, with 11 million patients died, accounting for 19.7% of all global deaths [2]. According to the World Health Organization (WHO), sepsis and sepsis-induced shock are serious public health problems, and statistics declares that improved prevention, diagnosis, and clinical management of sepsis and treatment are priority of healthcare worldwide [3]. According to a recent systematic review, the mortality rate from sepsis is 27% [4]. Early intervention for “high-risk” sepsis is deemed critical for better clinical outcomes. Accordingly, new strategies are needed to identify patients at risk for sepsis mortality more reliability so as to personalize treatment strategies.

Ferroptosis, a type of iron-dependent programmed cell death, has lately attracted increasingly attention due to its intensive implication in inflammatory conditions [5], and research on the mechanisms underlying the link between cellular ferroptosis, infection, and inflammation is growing fast. Ferroptosis is an inflammatory and immunogenic condition that promotes the release of pro-inflammatory cytokines and contributes to the pathogenesis of sepsis [6]. Several studies have found that ferroptosis plays a role in immune cell function. The number of immune cells, such as macrophages, T cells, and B cells, is reduced by ferroptosis. Immune cells recognize iron-depleted dead cells and initiate a chain reaction of inflammatory or specific immune responses [7]. Therefore, ferroptosis-associated genes (FAGs)have been recognized as promising diagnostic markers and potential therapeutic targets for sepsis [8], and prognosis models for the sepsis of children [9], or for sepsis-induced organ failure have been reported [10]. However, the value of FAGs in sepsis subtype identification and prognostic prediction remains unclear.

In this study, we identified the FAG-based molecular subtypes, created a prognosis model for sepsis patients, and investigated the relationship between FAGs and the immune status. We evaluated the robustness of the model using an external validation set and identified one hub gene in the model. Our findings shed light on new mechanisms and ferroptosis targets, paving the way for effective ferroptosis-targeted therapy for sepsis patients.

## 2. Materials and Methods

### 2.1. Data Sources and Preprocessing

The Gene Expression Omnibus (GEO) database provides complete RNA expression patterns and survival information of 603 sepsis patients. After excluding patients who had missing information on key predictors: including age, gender, and survival outcomes, a total of 581 cases were enrolled in this study. The GSE65682 included 479 sepsis patients who were randomly assigned to either the training (n = 360) or the test (n = 119) groups. The GSE95233 datasets (n = 102), served as the external validation cohort for the purpose of assessing the predictability and robustness of the ferroptosis-associated predictive risk model. The relevant grouping information and clinicopathological characteristics are shown in Table 1.

### 2.2. Identification of Ferroptosis-Associated Subtypes

The FerrDB database (http://www.zhounan.org/ferrdb/current/), accessed on 28 July 2022, contained 487 unduplicated ferroptosis-related genes, including markers, drivers, and suppressors. Together with survival data, we identified 57 of these genes associated with survival by univariate Cox regression analysis (*p* < 0.05). Based on their expression profiles, consensus clustering was performed to identify molecular subtypes associated with ferroptosis via the “ConcensusClusterPlus” package in R software. The parameters in this method were pam algorism with Manhattan distance, and sampling was performed 1000 times. The principal component analysis (PCA) and t-distributed stochastic neighbor embedding analysis were performed to assess the classification of the two clusters.

### 2.3. Construction and Validation of a Ferroptosis-Based Risk Model

We used the least absolute shrinkage and selection operator (LASSO) regression algorithm to develop a prognostic model. This algorithm eliminates confounding variables by penalizing the coefficients of the variables, screening them more rigorously while reducing or eliminating multicollinearity. We performed ten-fold cross validation and selected the lambda value when the partial likelihood deviation reached a minimum. All these analyses were performed in the R package “glmnet”. Finally, our model contained 14 of the 57 FAGs. The risk score was calculated based on the linear combination of the regression coefficient (β) from LASSO regression multiplied by gene expression levels (risk score = ∑βi*RNAi). Patients were classified into high-risk or low-risk group based on their risk scores and maximumly selected rank statistics. A time-dependent receiver operating characteristic (ROC) curve analysis was performed to assess the model’s prediction accuracy. Cox regression analysis was used to examine independent prognostic significance.

### 2.4. Construction and Validation of a Predictive Nomogram

To screen for independent predictors of the sepsis outcome, we analyzed risk scores and clinicopathologic characteristics, including age, sex, and site of infection in sepsis, by univariate and multivariate Cox regression analysis. Independent variables from multivariate Cox regression analysis, yielded a nomogram of 1-, 14-, and 27-day survival probability for sepsis patients. The calibration and ROC curves were plotted to measure the clinical benefits of the nomogram.

### 2.5. Immune Analysis

We performed the ESTIMATE and CIBERSORT methods to determine the relative proportions of 22 infiltrating immune cells and their relationships with the risk score.

### 2.6. Gene Set Variation Analysis (GSVA) and Functional Enrichment Analysis

Gene set variation analysis (GSVA) can be used to estimate the relative enrichment of a gene set of interest over a sample population [11]. We downloaded gene sets from the Molecular Signatures Database (http://software.broad-institute.org/gsea/msigdb), accessed on 28 July 2022, and then used GSVA package in R language to ascribe the signaling pathway variation scores to the gene sets to evaluate their biological functions.

### 2.7. Weighted Gene Co-Expression Network Analysis (WGCNA)

Weighted correlation network analysis (WGCNA) is an extremely effective algorithm. It can uncover modular genes related to disease characteristics and targeted therapies and find genetic modules with highly comparable expression. To find the hub genes among the 14 model genes, we used the complete gene expression data GSE65682 to create gene modules using WGCNA and subsequently found the modules most associated with clinical phenotypic risk factors. The hub genes were the intersection of 14 models and module genes. The soft threshold was calculated using the function “power Estimate”. The gene modules were built as follows: the weighted adjacency matrix was transformed into a topological overlap matrix (TOM) to analyze the network connectivity, and the hierarchical clustering method was used to construct the clustering tree structure of the TOM. Different branches of the clustering tree represented different gene modules, and different colors represented different modules. Tens of thousands of genes were grouped into modules according to their expression patterns.

### 2.8. Statistical Analysis

Survival curves were generated by the Kaplan–Meier method and were compared by the log-rank test. The multivariate Cox proportional hazards regression was used to identify independent prognostic factors. The time-dependent area under the curve (AUC) of ROC curves for survival variables was conducted by the “timeROC” package. Correlations between two continuous variables were assessed by Pearson’s correlation coefficients. All statistical analyses were performed in R software (v4.0). All statistical tests were two tailed, and *p* < 0.05 was considered statistically significant.

## 3. Results

### 3.1. Identification of Prognosis-Associated Genes in Sepsis

The flow diagram (Figure S1) in Supplementary File S1 illustrates the detailed study procedure. A total of 487 FAGs were downloaded from the ferrDB after removing duplicates, from which, 57 prognostic genes were identified by univariate Cox regression (*p* < 0.05; Supplementary File S2) based on gene expression profiles from the GSE65682 dataset.

### 3.2. Identification of Ferroptosis-Associated Molecular Subgroups

**Figure 1:**
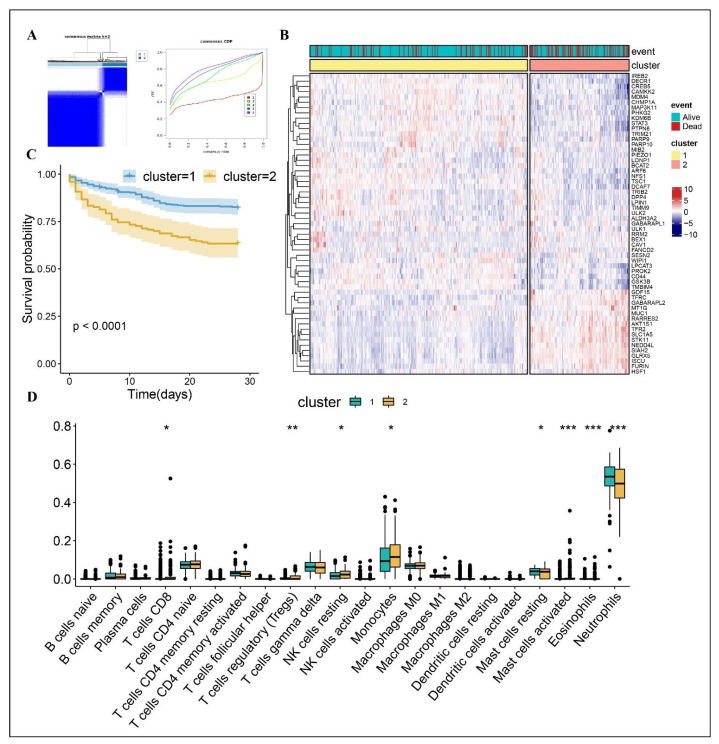
Consensus clustering and the different immune profiles between two clusters. (**A**) Consensus matrix heatmap indicating that the optimal value for consensus clustering is K = 2. (**B**) Heatmap visualizing the different expression pattern of the 57 FAGs in the two clusters. (**C**) Survival curve of the patients in the two clusters. (**D**) CIBERSORT analysis in the two clusters. * *p* < 0.05; ** *p* < 0.01; *** *p* < 0.001.

We divided 479 patients in the GSE65682 into different ferroptosis-associated molecular subgroups using consensus clustering and expression patterns of 48 prognosis-associated FAGs. As the clustering variable (k) increased, two clusters (k = 2) were found to be the most acceptable for consensus clustering (Figure 1A). Consequently, the number (k) was selected as two to get two molecular subtypes. The (PCA) and t-distributed stochastic neighbor embedding analysis (t-SNE) both revealed substantial distinction in the two cluster subgroups (Figures S2 and S3), with 329 patients in Cluster 1 and 150 in Cluster 2. Heatmap visualization detected significant differences in the expressions of the 57 FAGs between the two clusters (Figure 1B). Patients stratified into cluster 2 subtypes were associated with high mortality (log rank test, *p* < 0.0001) (Figure 1C). In addition, the CIBERSORT method was utilized to examine the differences in immune cell infiltration between the two clusters. Figure 1D showed that cluster 1 had higher infiltration levels of CD8 T cells, Tregs cells, monocytes, mast cells resting, mast cells activated and eosinophiles, and a lower infiltration level of neutrophiles. The findings suggested significant heterogeneity in sepsis patients, and FAGs played an important role in sepsis.

### 3.3. Functional Enrichment Analysis of Two Clutters

GSVA was performed to investigate the biological behaviors of the two ferroptosis clusters. Go enrichment analysis showed that ferroptosis cluster 1 was associated with positive regulation of iron-ion transmembrane transport, axon diameter regulation, and carbon dioxide transport, whereas ferroptosis cluster 2 was associated with positive regulation of interleukin 23 production, ccr4 not core complex, and negative regulation of viral genome replication by host (Figure S4). In addition, cluster 1 was associated with natural killer cell mediated cytotoxicity, the b cell receptor signaling pathway, and the fc epsilon ri signaling pathway, whereas ferroptosis cluster 2 was associated with porphyrin and chlorophyll metabolism, proximal tubule bicarbonate reclamation, and riboflavin metabolism, according to KEGG enrichment analysis (Figure S5).

### 3.4. Construction and Validation of a Prognosis-Associated Risk Model Composed of 14 FAGs

**Figure 2:**
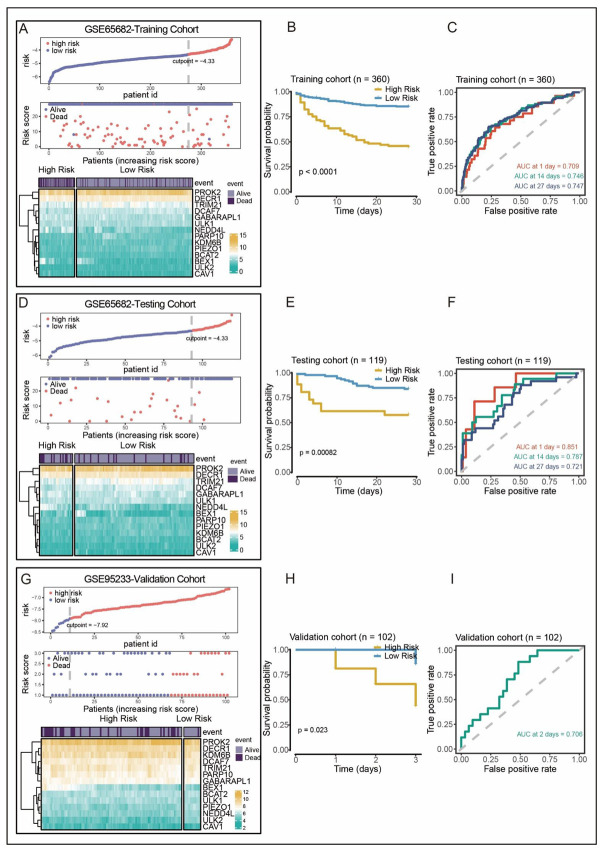
Construction of the risk model in the GSE65682 training cohort and validation of the risk model in the GSE95233 test cohort. (**A**,**D**,**G**) Distribution of the risk score, survival status, and gene expression of 14 FAGs in the GSE65682 training cohort (**A**), GSE65682 test cohort (**D**) and GSE95233 cohort (**G**). (**B**,**E**,**H**) Kaplan–Meier curves of OS of patients in the high- and low risk groups in the GSE65682 training cohort (**B**), GSE65682 test cohort (**E**) and GSE95233 cohort (**H**). (**C**,**F**,**I**) ROC curves for predicting the 1/14-day overall survival in the GSE65682 training cohort (**C**), and ROC curves for prediction the 2-day OS in the GSE95233 cohort.

We constructed a ferroptosis-associated prognostic risk model base on 14FAGs, which were identified by univariate LASSO Cox regression analysis. The 479 GEO patients were randomly assigned (4:1 ratio) into the training group (n = 360) or the test (n = 119) to ensure the similar gene expression patterns between the two groups. Each patient’s risk score was calculated using the risk model as follows: ULK2∗ −0.722782) + ULK1∗ (0.007177) + TRIM21∗ (−0.029375) + PROK2∗ (−0.069122) + PIEZO1∗ (−0.356316) + PARP10∗ (−0.109444) + NEDD4L∗ (0.116562) + KDM6B∗ (−0.117215) + GABARAPL1∗ (0.208982) + DECR1∗ (−0.156404) + DCAF7∗ (−0.090594) + CAV1∗ (0.090246) + BEX1∗ (0.089132) + BCAT2∗ (−0.073745). The patients were classified into the high-risk or low-risk group according to maximumly selected rank statistics. Figure 2A depicts the distribution of risk score, survival status, and expression of the 14 FAGs in the training cohort. It was discovered that the expression of genes with hazard ratios greater than one, such as ULK1, NEDD4L, GABARAPL1, CAV1, and BEX1 were higher in the high-risk group, while the expression of genes with hazard ratios less than one, such as ULK2, TRIM21, PROK2, PIEZO1, PARP10, KDM6B, DECR1, DCAF7, and BCAT2, were higher in the low-risk group. Additionally, Kaplan–Meier curves demonstrated that the sepsis patients in the high-risk group had considerably lower OS. The AUC values of ROC curves for 1-, 14-, and 27-day OS were 0.709, 0.746, and 0.747, respectively (Figure 2C), indicating an excellent predictive performance of the prognosis-associated risk model.

Next, the prediction performance and robustness of the prognosis-associated risk model were evaluated using the GSE65682 test cohort as an internal validation cohort and the GSE95233 dataset as an external validation cohort. Following the same formula, consistent results were obtained from the test cohort, which proved the robustness of the risk model. The risk score distribution and gene expression profiles are displayed in Figure 2D. The OS was considerably worse in the high-risk group than in the low-risk group (Figure 2E). The AUC values for 1-, 2-, and 3-day OS were 0.851, 0.787, and 0.721, respectively (Figure 2F). The external validation cohort only provided 3-day survival data, and the AUC of the ROC values for 2-day OS were 0.706, suggesting that our prognostic model had strong prognostic value. This results in the external validation cohort coincided with the training cohort results. According to the risk score distribution and gene expression profiles in Figure 2G, patients with higher risk scores showed a worse OS (Figure 2H). These findings revealed that the risk model was capable of accurately predicting OS.

### 3.5. Clinical Correlations and Independent Prognosis Analysis of Risk Score

**Figure 3:**
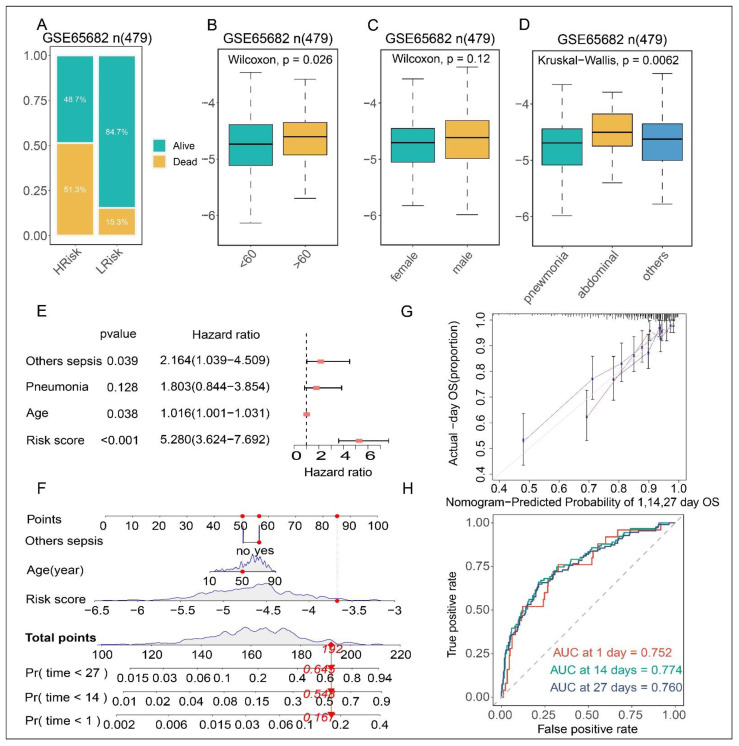
Clinical correlations of risk score and development of the nomogram in the GSE65682 dataset. (**A**–**D**) Relationships between the risk score and clinicopathological characteristics (age, gender, Infection site). (**E**) Multivariate analyses revealed the risk score was an independent prognostic factor for sepsis patients. (**F**) Nomogram for predicting the 1-, 14-, and 27-days OS of sepsis patients. (**G**) Calibration curves of the nomogram for OS prediction at 1-, 14-, and 27-days. (**H**) ROC curves predicting 1-, 14-and 27- days OS. Draw a line straight upward to the point’s axis to give a score of each variable, and finally add the score of the three variables (sepsis infectious site, age, and risk score) to obtain the total score. The probability of survival is obtained by drawing a vertical line down the total score. Others sepsis = Sepsis, except for pneumonia and abdominal sepsis; Age = age in years; Risk score = risk score calculated by 14-FAG model; Pr = survival probability.

To further validate the importance of risk score in clinical practice, its correlation with clinicopathological features was examined. Figure 3A shows that the mortality rate in the high-risk group was higher than that in the low-risk group. In addition, high risk scores were associated with advanced age and abdominal sepsis (Figure 3B–D, Kruskal–Wallis rank sum tests, *p* < 0.05). However, there was no statistically significant difference in risk scores between male and female. Multivariate Cox regression suggested that risk score was independent risk factor for OS of sepsis patients (HR = 5.280, *p* < 0.001). Subsequently, we constructed a nomogram by combining the risk scores with all relevant clinical characteristics to predict the prognosis of sepsis patients to guide clinical decision making. Nomogram indicated that the risk score had more significant effect on prognosis, than other clinical characteristics (Figure 3F). Moreover, calibration and ROC curves for the predicted 1-, 14-, and 27-day survival rates were created. The results revealed a high degree of consistency between the survival prediction curve and the reference curve, as well as a high AUC value, implying that the model performed well in survival prediction.

### 3.6. Relationship between Risk Score and Immune Cell Infiltration

**Figure 4:**
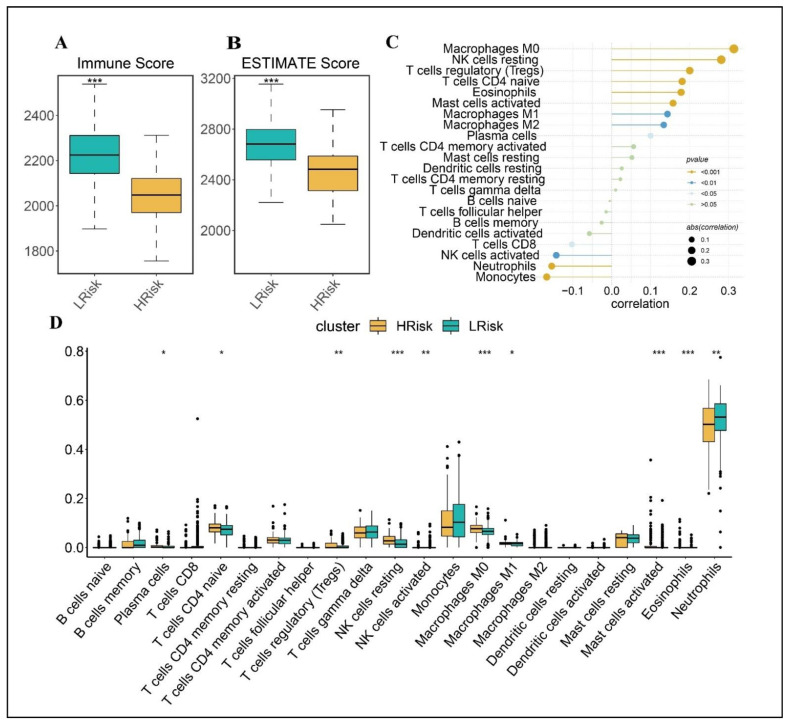
The different immune profiles between the low- and high- risk groups in the GSE65682 dataset. Two risk groups were divided based on the median risk score. (**A**) Immune score. (**B**) ESTIMATE score. (**C**) Correlation between risk score and (**D**) immune cell content. *p* values were showed as: ns not significant; * *p* < 0.05; ** *p* < 0.01; *** *p* < 0.001.

Sepsis treatment and prognosis are influenced by immune status. To detect correlations between risk scores and levels of immune cell infiltration, we employed two methods: ESTIMATE and CIBERSORT. The ESTIMATES results suggested that the patients in the high-risk group had significantly lower immune scores and estimate scores than the patients in the low-risk group (Figure 4A,B). By digging deeper into the connections between the risk score and immune cell content, it was found that the risk score was positively associated with macrophages M2, T cells CD4 regulatory, T cells CD4 naive, eosinophils, and activated mast cells, and was negatively associated with monocytes and neutrophils. Furthermore, the most infiltrating immune cells differed in two groups according to CIBERSORT analysis: the high-risk group had significantly higher abundances of T cells naive, T cells regulatory, NK cells resting, NK cells activated, macrophages M0, M1, mast cells activated, and eosinophils, whereas the low-risk group had a higher abundance of neutrophils.

### 3.7. Screening for Hub Genes in the Risk Model by WGCNA

**Figure 5:**
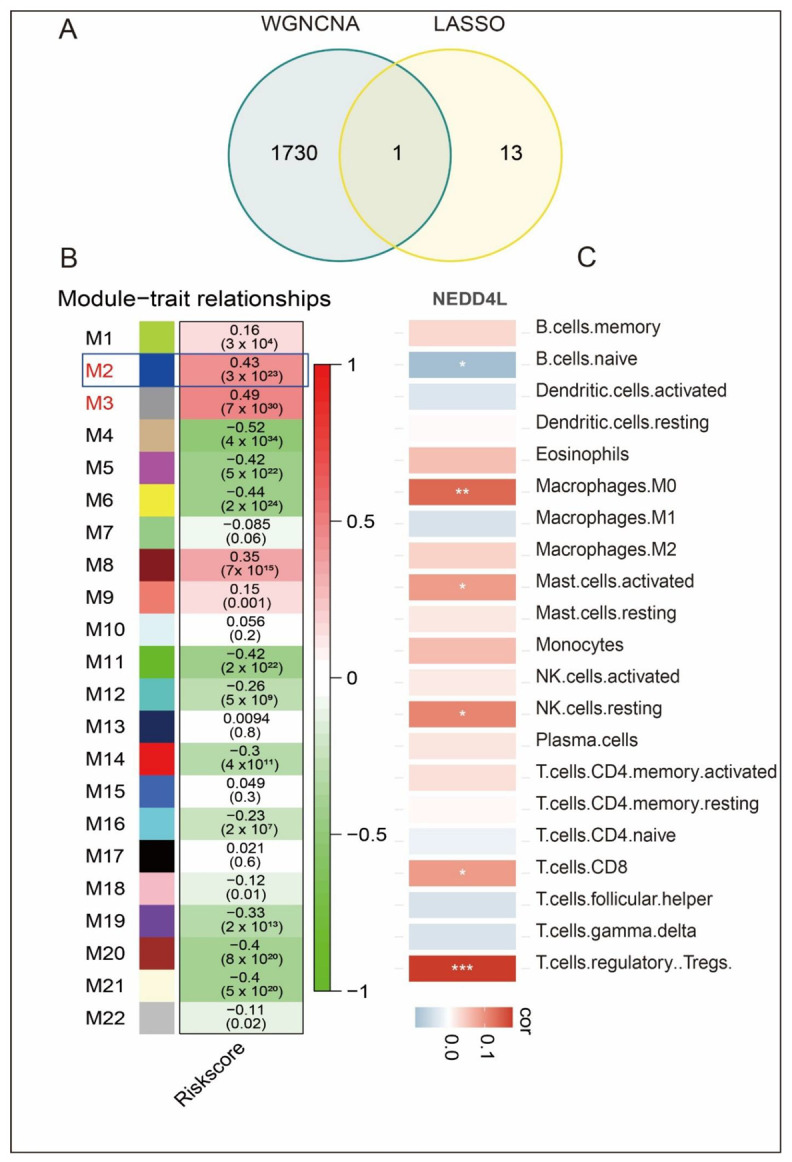
Identification of one hub gene and prediction of their relationship with immune cell. (**A**) Venn diagram analysis showed that the overlap of WGCNA analysis and LASSO model led to one hub gene being identified: NEDD4L. (**B**) Heatmap of model trait relations. (**C**) Prediction of correlations between hub genes and immune cells. * *p* < 0.05; ** *p* < 0.01; *** *p* < 0.001.

We used the WGCNA to construct co-expression modules and identify modules associated with risk score. The soft-threshold was set to seven to build a scale-free network (Supplementary File S2: Figure S6). Then, an adjacency matrix was transformed it into a topological overlapping matrix (TOM), based on which a total of 22 modules were generated. The expression profile of each module was summarized by eigengenes (MEs) and was given a numeric identifier (“M” + number) (Figure 5B). Correlation analyses between the modules and risk score showed that the M3 module had the highest correlation (cor = 0.49, *p* = 2 × 10^−30^), followed by the M2 module (cor = 0.43, = 6 × 10^−23^). To further verify hub genes among the 14 model genes, the overlapping genes between the M3 module and the 14 model genes were screened and no overlapping was found. Then the overlapping genes between the M2 module and the 14 module genes were screened, and one hub gene was identified: NEDD4L (Figure 5A,B). Finally, we investigated the relationship between NEDD4L and immune infiltrating levels. According to the findings, NEDD4L is related to macrophages M0, mast cells activated, NK cells resting, T cells CD8 and T cells regulatory (Figure 5C). These findings suggest that NEDD4L expression levels are associated with the prognosis of sepsis and immune status.

## 4. Discussion

Sepsis patients’ clinical and biological heterogeneity has long hampered the efforts in developing effective therapies [12]. So, there is an urgent need for a biomarker-based risk stratification model to identify patients with a high risk of death [13]. In this study, we identified two subtypes of sepsis associated with ferroptosis, and detected significant differences in prognosis and immune status between two subtypes. We also developed a prognostic model based on 14 FAGs, which demonstrated excellent predictive performance. Finally, we identified one hub genes among the model genes using WGCNA analysis.

We identified two ferroptosis-associated clusters, which presented significant different prognosis: The prognosis of group 2 was worse than that of group 1. The two clusters had different immune cell components, suggesting that immune cells such as neutrophils, monocytes, T cells regulatory, and mast cells play a role in the development of inflammatory response and the inflammatory state of the patient. Consistent with previous studies, lowered abundances of neutrophils [14], NK cells [15], and M1 macrophages [16] were associated with increased mortality in sepsis patients. Regulatory T cells (Tregs) can actively suppress immune response, resulting in septic immune dysfunction and increased mortality [17].

In the training cohort, we performed a univariate cox and lasso regression analysis to further evaluate the prognostic value of FAGs and constructed a prognosis model. We created a risk model with 14 FAGs, which performed well in the training, test, and external validation cohorts. The prognostic nomogram was proven robust for risk management in sepsis patients after combining all significant clinical characteristics. The above results indicate that the risk model is clinically applicable. In the view of the close connections, we investigated the differences in immune status between the high-risk and low-risk groups using the ESTIMATE and CIBERSORT methods. The high-risk group had a poorer prognosis, lower immune scores, and lower estimate scores than the low-risk group. This finding also suggests that FAGs are involved in the dysregulated response.

To further narrow down the 14 FAGs and identify hub genes, a WGNA analysis was performed, and one hub gene named NEDD4L, was detected by overlapping the genes in the M2 module with 14 FAGs. NEDD4L, or neural precursor cell expressed developmentally downregulated 4-like is an E3 ubiquitin ligase [18]. NEDD4L directly binds many iron channels, among which, the epithelial sodium channel (ENaC) is the mostly studied one [19]. ENaC-mediated sodium transport is essential for the proper functioning of multiple organs. For example, in the kidneys, it reabsorbs sodium and thus maintains the body’s blood pressure [20], and in the lungs, it removes fluid from the alveolar cavity and thus facilitates gas exchange [21]. NEDD4L accelerates the degradation of the cardiac sodium channel Nav1.5 through ubiquitination in the heart, which is associated with heart failure [22]. Combined our findings with the function of NEDD4L in various organs, NEDD4L may be involved in multi-organ failure due to sepsis, but further research is needed to validate this.

Although the clinical significance of NEDD4L in sepsis appears promising, our study has a few limitations. First, comprehensive experimental validation is required to assess the predictive value of NEDD4L. Second, the clinical and molecular characteristics in the GEO database were inadequate since prognostic factors, such as infectious agents of sepsis and background diseases were not included. Third, the significance of NEDD4L needs to be further validated in a prospective multicenter cohort on retrospective samples.

## 5. Conclusions

In this study, two ferroptosis-associated subtypes were identified, and a FAG-based risk model was constructed. The model was proven robust for predicting the prognosis of sepsis patients. Then a hub gene, namely NEDD4L was identified among 14-FAG model. Our study provides a new insight into potential molecular targets to combat sepsis and contributes to the basis of further research on FAGs as well as the underling mechanism in the pathogenesis of sepsis.

## Figures and Tables

**Table 1:** Clinicopathological characteristics of the sepsis cases in GSE65682 and GSE95233.

Characteristic	GSE65682			GSE95233 Validation Cohort (n = 102)
	All GSE65682 (n = 479)	Training Cohort (n = 360)	Testing Cohort (n = 119)	
Age (year), n%				
<60	277 (40.4)	143 (39.7)	50 (42.0)	42 (41.2)
>60	408 (59.6)	217 (60.3)	69 (58.0)	60 (58.8)
Gender, n%				
Male	288 (42.0)	204 (56.7)	68 (57.1)	36 (35.3)
Female	397 (58.0)	156 (43.3)	51 (42.9)	62 (60.8)
Human	0 (0.0)	0 (0.0)	0 (0.0)	4 (3.9)
OS status, n%				
Alive	365 (76.2)	272 (75.6)	93 (78.2)	34 (33.3)
Dead	114 (23.8)	88 (24.4)	26 (21.8)	68 (66.7)

OS status: overall survival status.

## Data Availability

All data can be available from the public database: Gene Expression Omnibus (GEO, available at: https://www.ncbi.nlm.nih.gov/geo/), accessed on 28 July 2022, database GSE65682 and GSE95233.

## Supplementary Materials

The following supporting information can be downloaded at: https://www.mdpi.com/article/10.3390/biom12101479/s1, Supplementary File S1, Figure S1: Flow chart of the data analyzing; Supplementary File S2: significant ferroptosis gene by univariate Cox regression. Supplementary File S3, Figure S2: principal component analysis of tow clusters; Supplementary File S3, Figure S3: tSNE analysis of tow clusters; Supplementary File S3, Figure S4: Go enrichment analysis via gsva; Supplementary File S3, Figure S5: Kegg enrichment analysis via gsva; Supplementary File S3, Figure S6: The soft-threshold to build a scale-free network.
