# Supplementary material for: Identification of Molecular Subtypes and a Novel Prognostic Model of Sepsis Based on Ferroptosis-Associated Gene Signature
Source: Biomolecules. 2022 Oct 14;12(10):1479. doi: 10.3390/biom12101479 (PMC9599462; doi:10.3390/biom12101479)
Supplement: Supplementary file 1 [file biomolecules-12-01479-s001.zip › File S3. s3_divid spesis patiens into two cluster.pdf]

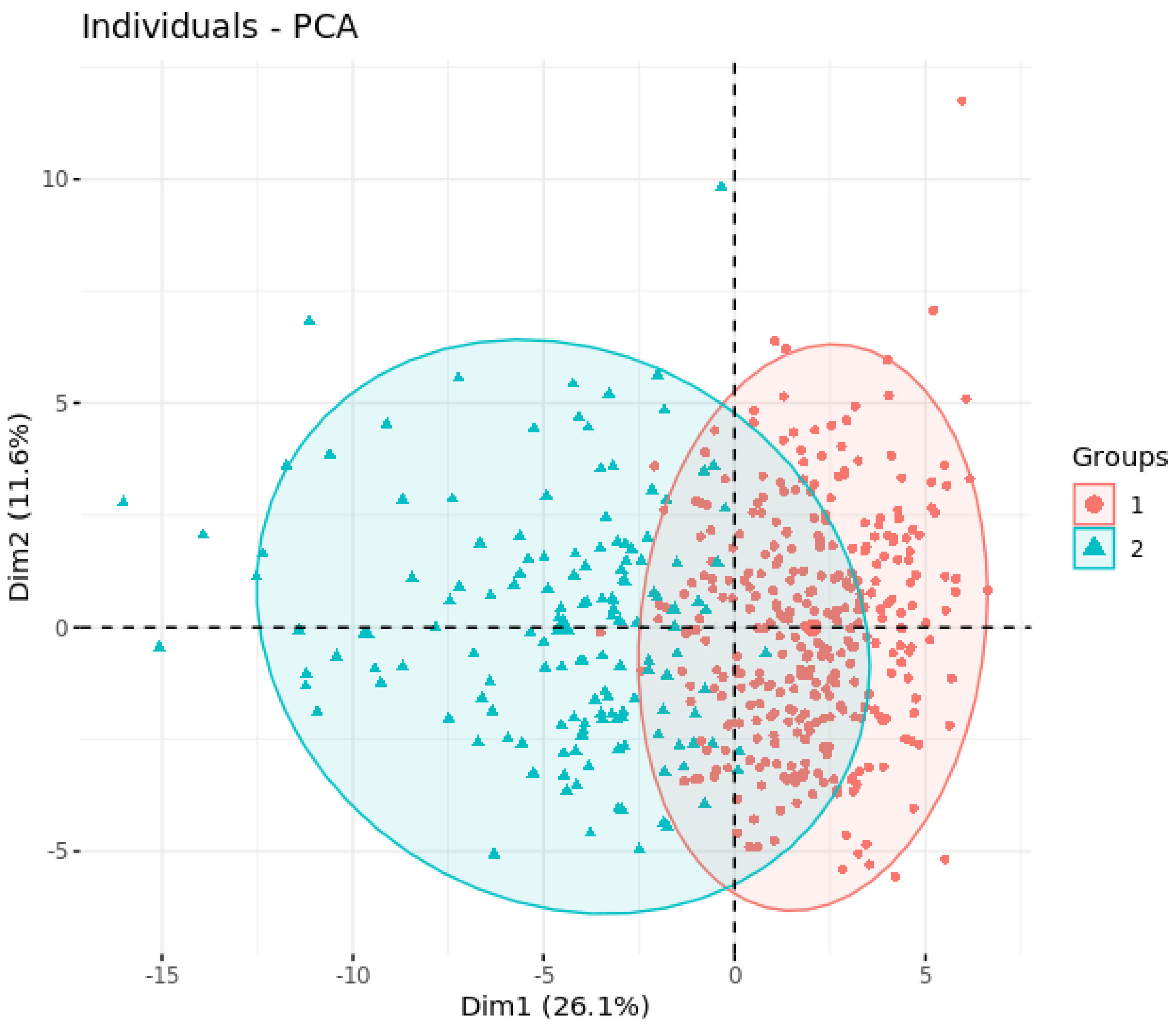

Figure S2: principal component analysis of tow clusters.

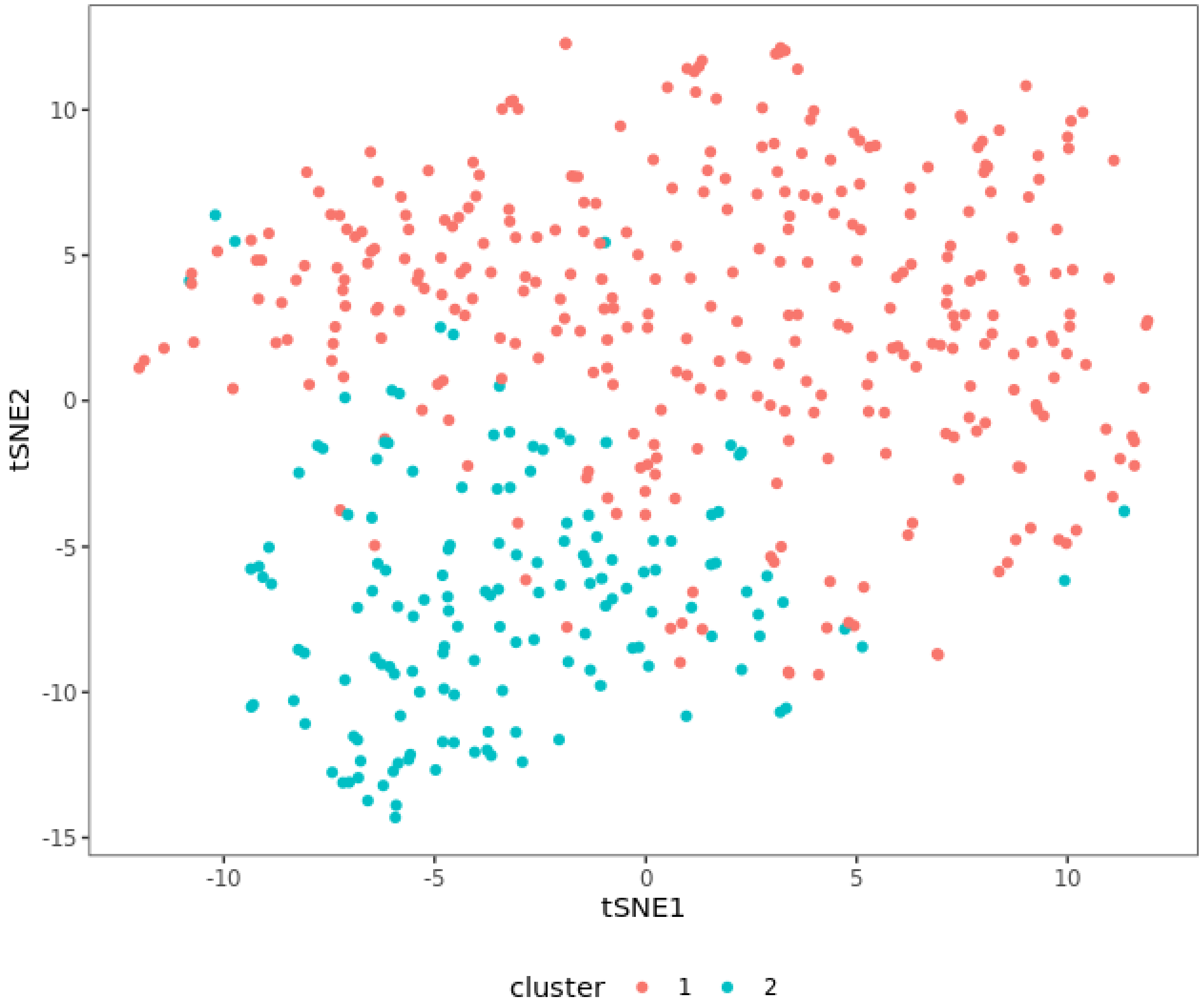

Figure S3: tSNE analysis of tow clusters.

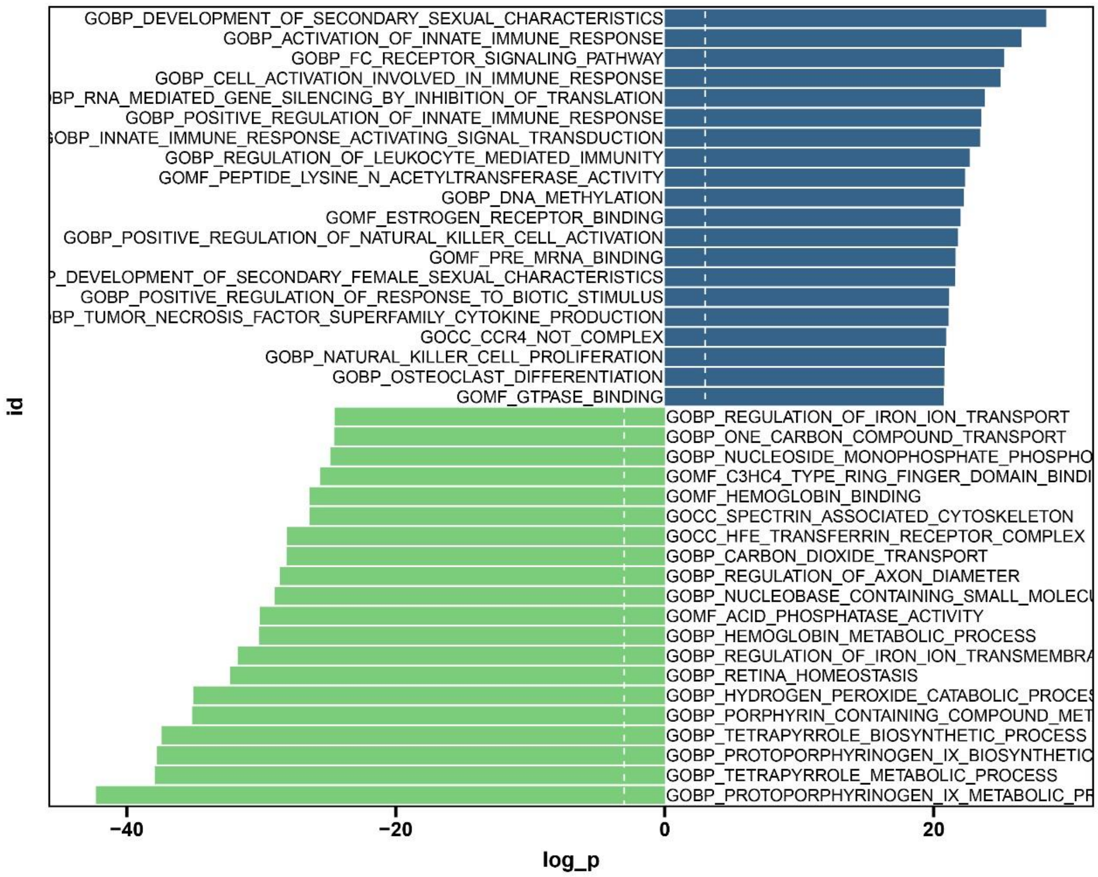

Figure S4: Go enrichment analysis via gsva.

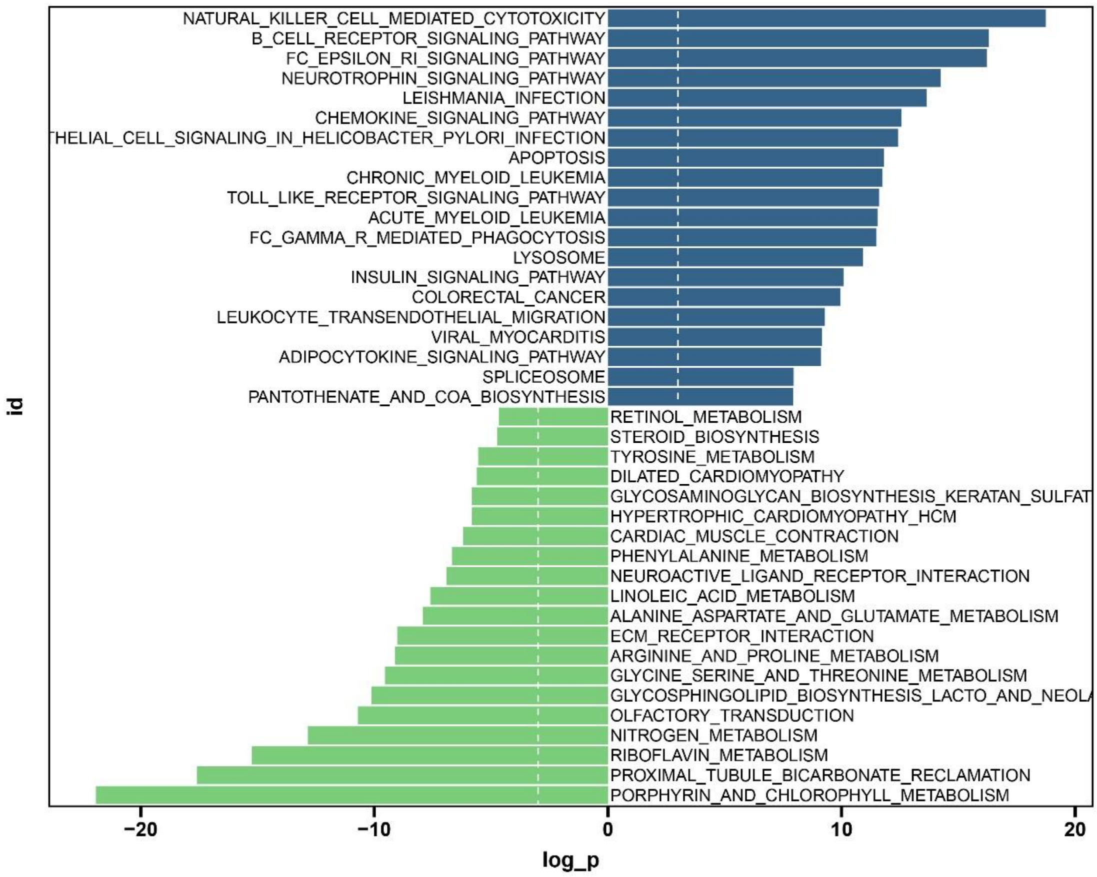

Figure S5: Kegg enrichment analysis vi gsva.

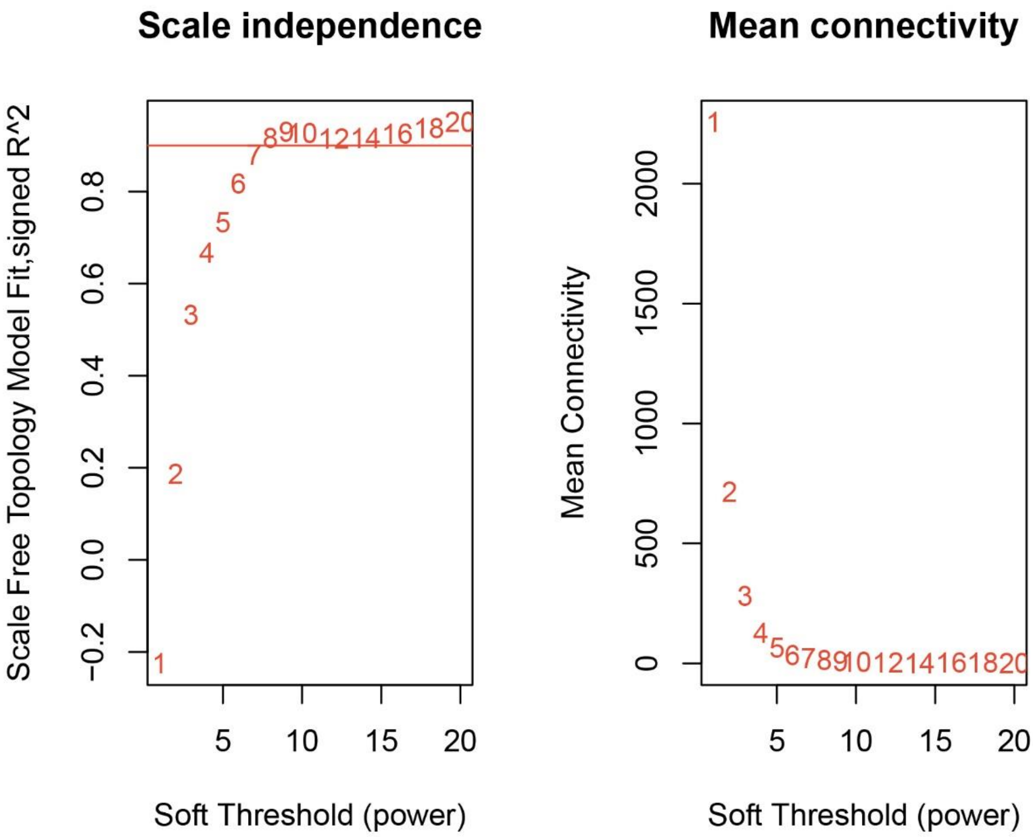

Figure S6: The soft-threshold to build a scale-free network.
