# Supplementary figures and images for: Identification of Molecular Subtypes and a Novel Prognostic Model of Sepsis Based on Ferroptosis-Associated Gene Signature
Source: Biomolecules. 2022 Oct 14;12(10):1479. doi: 10.3390/biom12101479 (PMC9599462; doi:10.3390/biom12101479)

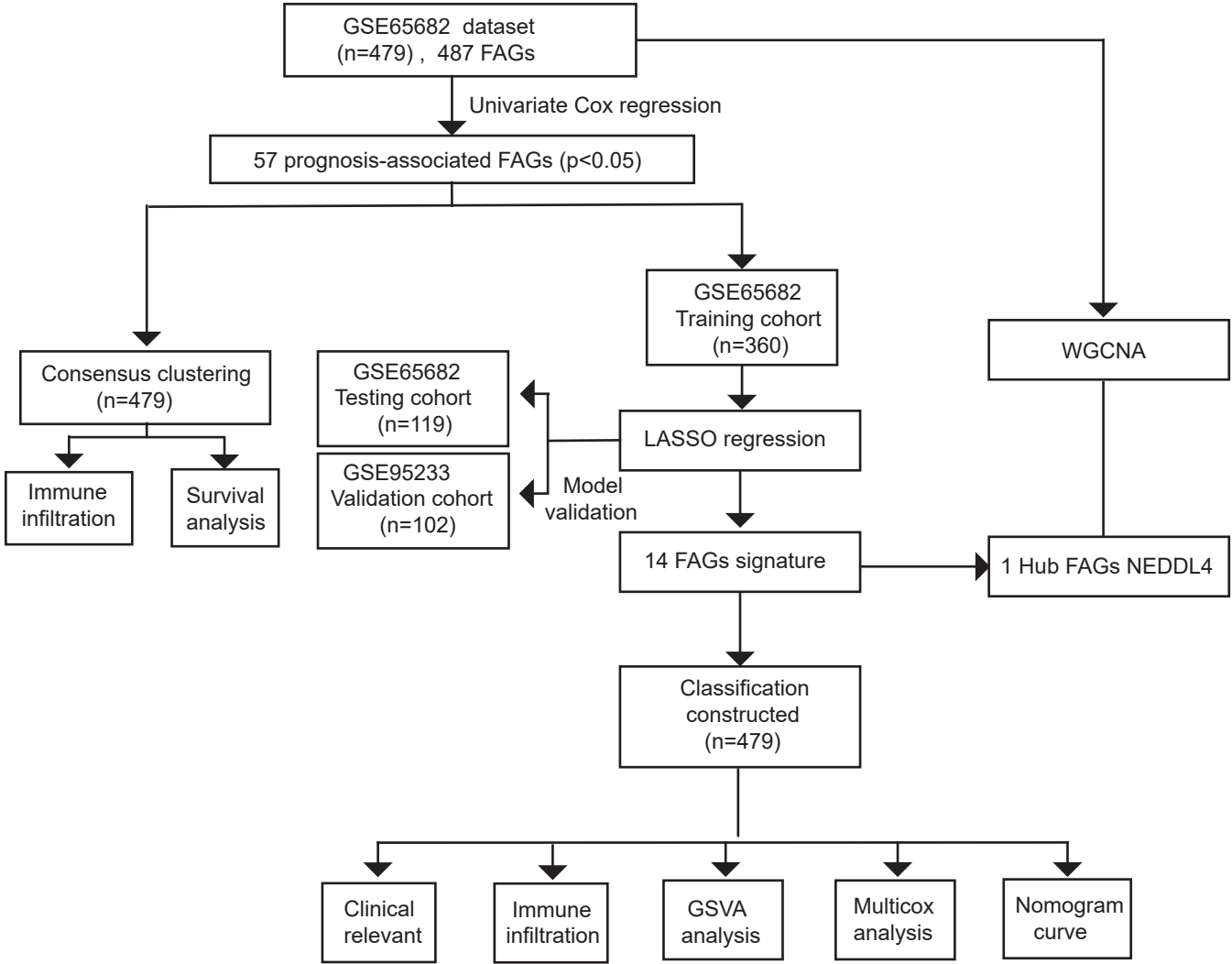

**Figure S1.** Flow chart of the data analyzing process. GSE65682 divides training set and testing set by 4:1

Supplement: Supplementary file 1 [file biomolecules-12-01479-s001.zip › File S1. Figure S1. Flow chart of the data analyzing.pdf]
